# Apoptosis in prostate carcinoma tissue: The role of caspase-3, caspase-1, and alkaline DNase activity

**DOI:** 10.5937/jomb0-57574

**Published:** 2025-09-05

**Authors:** Andrej Veljkovic, Jovan Hadzi-Djokic, Gordana Kocic, Xiaobo Li, Stefanos Roumeliotis, Dušan Sokolović, Aleksandra Klisic

**Affiliations:** 1 University of Nis, Faculty of Medicine; 2 Serbian Academy of Sciences and Arts; 3 Harbin Medical University, Harbin, China; 4 1st Department of Internal Medicine, AHEPA Hospital, School of Medicine, Aristotle University of Thessaloniki, Greece; 5 University of Montenegro, Faculty of Medicine, Podgorica, Montenegro

**Keywords:** prostate cancer, caspase-3, caspase-1, alkaline DNase, karcinom prostate, kaspaza-3, kaspaza-1, alkalna DNaza

## Abstract

**Background:**

Prostate glandular tissue maintains a delicate balance between cellular proliferation and programmed cell death (apoptosis), ensuring the preservation of normal glandular architecture in healthy individuals. Disruption of this equilibrium - whether due to excessive proliferation or impaired apoptotic mechanisms - can contribute to the initiation and progression of prostate cancer. The objective of this study was to evaluate the expression and activity of caspase-3, caspase-1, and alkaline deoxyribonuclease (DNase) in prostate cancer tissue and tumour-adjacent tissue in comparison to clinically healthy prostate tissue. The aim was to determine whether alterations in these parameters could serve as early biomarkers for the transformation of surrounding tissue into a precancerous phenotype.

**Methods:**

The concentration of caspase-3 and caspase-1, as well as the activity of alkaline DNase, were examined in prostate tissue samples, including cancerous tissue, adjacent tissue near the tumour, and surrounding healthy tissue.

**Results:**

The results revealed a significant reduction in caspase-3 levels in cancerous tissue (p<0.05), with an even more pronounced decrease in the adjacent peritumoural tissue (p<0.05). In contrast, caspase-1 levels were markedly elevated in both cancerous tissue (p<0.00001) and the surrounding non-malignant peritumoural tissue (p<0.0005). Similarly, alkaline DNase activity (both total and specific) was significantly increased in cancerous tissue (p<0.00001), with a moderate but statistically significant elevation in the tumour-adjacent tissue (p<0.000017) compared to control tissue.

**Conclusions:**

These findings suggest a disruption in the interplay between caspase-3 and alkaline DNase, potentially as a consequence of necrotic processes or enzyme release from inhibitory complexes. Furthermore, the increased expression of caspase-1 implies that inflammatory responses may play a role in tumourigenesis.

## Abstract (sr)

**Uvod:**

Žlezdano tkivo prostate odlikuje ravnoteža procesa proliferacije i programiranog odumiranja tkiva (apoptoze), koji su kod zdravih muškaraca u ravnoteži, što održava normalni volumen žlezde. Ukoliko dođe do poremećaja ova dva suprotna procesa, bilo na račun nekontrolisane proliferacije ili smanjene apoptoze, moguća je pojava karcinoma prostate. Cilj ovog istraživanja je da se ustanovi koncentracija kaspaze-3, kaspaze-1 i alkalne dezoksiribonukleaze (DNaze), u tkivu karcinoma prostate i u okolnom tkivu, u odnosu na klinički zdravo tkivo, kako bi se ustanovilo da li njihova ekspresija i aktivnost može biti rani marker alteracije okolnog tkiva u prekancerozni fenotip, u odnosu na zdravo tkivo.

**Metode:**

Koncentracija kaspaze-3 i kaspaze-1, kao i aktivnost alkalne DNaze, ispitani su u uzorcima tkiva prostate, uključujući kancerogeno tkivo, susedno tkivo u blizini tumora i okolno zdravo tkivo.

**Rezultati:**

Koncentracija kaspaze-3 pokazuje značajan pad u tkivu karcinoma (p<0,05), pri čemu je pad prisutan i u okolnom tkivu neposredno uz karcinom (p<0,0005). Aktivnost alkalne DNaze (totalna i specifična) u karcinomu prostate je bila značajno povećana (p<0,00001), a nešto manje u okolnom tkivu neposredno uz karcinom (p<0,000017) u odnosu na udaljeno tkivo.

**Zaključak:**

Dobijeni podaci pokazuju da se međuzavisnost kaspaze-3 i alkalne DNaze gubi ili zbog prisutne nekroze, ili zbog oslobađanja enzima iz kompleksa sa inhibitorom, dok porast kaspaze-1 implicira da zapaljenjska reakcija može da ima ulogu promotora kancerogeneze.

## Introduction

Prostate cancer (PC) is the leading cause of cancer- related mortality in men, with an increasing incidence. Despite continuous advancements in clinical, biochemical, histopathological, and radiological techniques for early detection and modern therapies, incidence is still growing [1]. Due to its highly aggressive nature, rapid proliferation, high metastatic potential, and resistance to therapy, a major challenge in elucidating the mechanisms of development of the PC. The high mortality rate is primarily attributed to metastasis in bone and lymphatic tissue. The tendency for rapid progression depends on both androgendependent and androgen-independent tumour growth [2]. Prognosis is determined by histopathological findings, including the Gleason score and specific tumour markers, primarily prostate-specific antigen (PSA) [3].

Among the known risk factors contributing to prostate cancer development are advanced age (with a significantly higher incidence after the age of 50), race (with a higher prevalence in African Americans), family history, diet (i.e., a diet rich in meat and dairy products), and chronic prostate infections [4] [5] [6] [7]. From the earliest insights into prostate cancer pathogenesis to the current level of understanding regarding its development, progression, and prognosis, there has been an ongoing effort to define and establish an ideal or earlier tumour marker that could have a significant impact on pathogenesis, diagnosis, and potential treatment [1] [2] [3].

Regarding the compartmentalization of prostate cancer within the prostate gland, the peripheral zone represents a typical localization for both carcinoma and inflammatory processes. Therefore, it is not surprising that chronic infections and inflammation frequently coexist with early carcinogenesis. The inflammatory process can promote tumour progression by inducing DNA damage and inadequate DNA repair, whether mediated by viruses, bacteria, harmful dietary substances, hormones, urine reflux, or as a result of an autoimmune response [4] [5].

As a gland, the prostate undergoes continuous proliferation and tissue apoptosis, ensuring a balance between these two opposing processes in healthy males. Those processes maintain normal glandular volume. If this balance is disrupted, the potential emergence of carcinoma is a likely consequence [8].

Apoptosis is a distinct form of programmed cell death that takes place during both normal physiological processes and pathological conditions. A defining feature of apoptosis is its confinement to individual cells or small cell groups, in contrast to necrosis, which involves the death of large cell populations and is typically accompanied by inflammation.

Alongside biochemical markers of apoptosis, a highly characteristic histomorphological pattern is also evident, marked by chromatin condensation caused by DNA fragmentation, cell shrinkage, and the subsequent formation of distinct apoptotic bodies. These apoptotic bodies are subsequently phagocytosed, thereby preventing an inflammatory response.

From a biochemical perspective, the process of apoptosis is characterized by the proteolytic activity of caspases. The active site of these enzymes contains cysteine, and they specifically cleave aspartate bonds within proteins – hence their name (Cysteine-ASpartate). In a healthy cell, these enzymes exist in an inactive form as procaspases, while their activation involves the formation of a heterotetramer consisting of two large and two small subunits. The activation of the caspase cascade occurs through a series of sequential reactions. Based on protein structure and activation mechanism, caspases can be divided into initiator and executioner caspases. Initiator caspases include caspase-2, -8, -9, and -10. Executioner caspases are caspase-3, -6, and -7. Other caspases (caspase-1, -4, -5, and -12) are structurally related to caspases but primarily play a role in the process of inflammation. Among the three known types of cell death (apoptosis, necrosis, and pyroptosis), pyroptosis is triggered by the activation of caspase-1 [9] [10] [11].

Caspase-1 is present in tissues in an inactive form, and in order to become functional, it is activated through association with the inflammasome complex. Inflammasomes are cytosolic multiprotein complexes that are released in response to external signals associated with pathogenic noxae (pathogen-associated molecular patterns – PAMP) and/or damage (damage-associated molecular patterns – DAMP), induced by immune cells. Immune cells can detect these stimuli through recognition receptors [12] [13]. Caspase-1 is a well-studied inflammatory caspase that plays a crucial role in the maturation of pro-inflammatory cytokines (pro-interleukin (IL)-1β and IL-18) into IL-1β and IL-18 [14].

Although the exact molecular mechanism of prostate cancer development associated with chronicinflammation is not entirely clear, it is believed that the main contributing factors are the production of inflammation-related cytokines, such as the IL-1 family, IL-6, and tumour necrosis factor-alpha (TNF-α), as well as the generation of reactive oxygen species (ROS). The released cytokines further amplify cellular damage, which serves as a significant stimulus for carcinogenesis [15].

The process of DNA degradation in cells involves a family of enzymes known as deoxyribonucleases (DNases). Alkaline DNase is the enzyme predominantly responsible for DNA degradation, which occurs in an alkaline pH environment. This family of various endonucleases with deoxyribonuclease activity is identified, but many proteins can also acquire DNase-like properties when pH conditions change. Degradation and fragmentation of nuclear DNA are common occurrences during apoptosis. It has been established that during apoptosis, a cell undergoes epigenetic reprogramming. In the D1 phase of programmed cell death, double-stranded DNA activation occurs, leading to DNA fragmentation, which significantly alters nuclear morphology in the F phase [16]. DNA fragmentation during apoptosis is considered to have a protectivefunction, as it reduces the likelihood of transmitting mutation-carrying genes from potentially active sites to the nuclei of healthy neighbouring cells [17].

Understanding the imbalance between apoptosis and proliferation is fundamental to revealingthe process of carcinogenesis. Therefore, this study aimed to determine the expression of caspase-3, caspase-1, and alkaline DNase in prostate cancer tissue and to assess whether their expression and activity could serve as early markers of tissue alteration toward a precancerous phenotype compared to healthy tissue.

## Materials and methods

The Ethics Committee of the Faculty of Medicine, University of Niš, approved the proposed study protocol, and all the patients provided informed consent (No. 12-8818-2/18). This pilot study was conducted at the University Clinical Center Niš, where 26 patients who underwent total prostatectomy were analyzed. The diagnosis was confirmed based on clinical symptoms, findings from digital rectal examination (DRE), and elevated PSA levels beyond the agespecific reference range.

### Tissue preparation

After radical prostatectomy, the next step was sampling, which involved excising sections of prostate cancer tissue, adjacent macroscopically healthy tissue, and macroscopically healthy tissue located at least 2 cm away from the tumour. This resulted in a total of 78 samples. The obtained samples were homogenized on ice, prepared as 10% homogenates, and frozen at -80°C until biochemical analyses were conducted.

### Enzyme analyses

The protocols used for determining caspase-3 and caspase-1 activity were based on the enzymelinked immunosorbent assay (ELISA) method. The tests were obtained from Elabscience (Catalog No. E-EL-H0017, USA) for caspase-3, with a detection range of 0.31–20 ng/mL, and Elabscience (Catalog No. E-EL-H0016, USA) for caspase-1, with a detection range of 78.13–5000 pg/mL.

The protocols used for DNase activity determination were optimized in the laboratory of the Department of Biochemistry and were previously published for tissue and cell culture samples [18] as well as for plasma samples [19]. The method is specific for nucleases and is based on spectrophotometric measurement of released acid-soluble nucleotides from the appropriate substrates (in this case, DNA), which are read at 260 nm after the precipitation of undegraded nucleic acids. The DNA substrate was obtained from Sigma-Aldrich, Germany.

Enzyme activity was expressed as total activity (IU/L homogenate) and specific activity (IU/g protein of fresh tumour tissue). Protein content in tissue homogenates was measured using the method by Popovic et al. [20].

## Results

**Figure 1:**
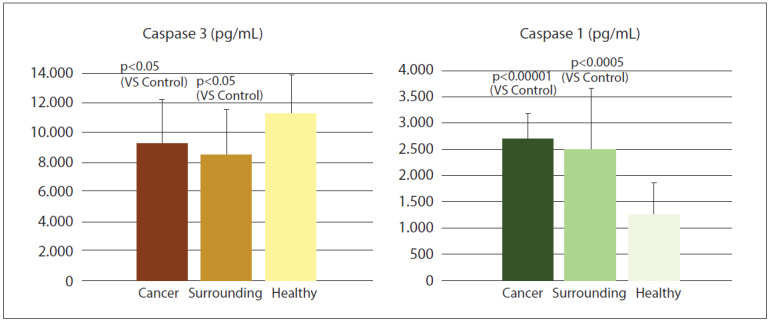
Concentration of caspase-3 and caspase-1 in prostate cancer, surrounding tissue adjacent to the carcinoma, and distant (healthy) prostate tissue.

**Figure 2:**
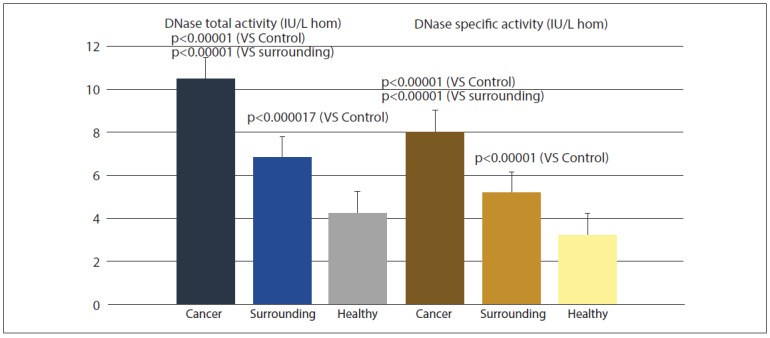
Alkaline DNase activity (total and specific) in prostate cancer, surrounding tissue adjacent to the carcinoma, and distant (healthy) prostate tissue.

The study results are presented in Table 1 and Figure 1 and Figure 2. Table 1 summarizes patient characteristics, including age, PSA levels, Gleason score, and TNM stage.

**Table 1:** Patient age and tumor marker values in prostate cancer.

Examined parameters	Arithmetic mean ± SD
Years of age	68.05±4.55<br>(range 35–77)
tPSA (ng/mL)	17.98±10.94<br>(range 5.66–36.47)
Gleason score	6.65±0.61<br>(range 6–8)
Tumour stage (TNM)	2.34±0.48<br>(range 2–3)

Figure 1 displays the concentrations of caspase-3 and caspase-1 in the samples. Caspase-3 levels show a significant decrease in cancerous tissue. Notably, the reduction is also present in adjacenttissue, where the decline appears even more pronounced. Caspase-1 levels are significantly elevated in both the cancerous tissue and the adjacent healthy tissue near the tumour.

Figure 2 shows the total and specific activity of alkaline DNase. DNase activity is significantly elevated in cancerous tissue compared to both adjacent and healthy tissue. However, DNase activity in adjacent tissue is also increased relative to healthy tissue.

## Discussion

In the conducted study, the concentration of caspase-3 and caspase-1, as well as the activity of alkaline DNase, were examined in prostate tissue samples, including cancerous tissue, adjacent tissue near the tumour, and surrounding healthy tissue. The diagnosis and staging of prostate cancer were verified through clinical symptoms, abnormal findings from digital rectal examination, elevated PSA levels, TNM staging – based on T (size of the primary tumour), N (spread to nearby lymph nodes), and M (distant metastases) – and Gleason score assessment (6 indicating low grade, 7 intermediate, and 8-10 high grade), according to histopathological findings. PSA is an example of a tissue-specific but not entirely tumour-specific antigen, widely used as a biomarker for the diagnosis and prognosis of prostate cancer. However, its specificity for cancer is not absolute, and it lacks high sensitivity in distinguishing highly aggressive cancer forms. The Gleason score is commonly used in the prognostic evaluation of prostate cancer [21] [22].

The concentration of caspase-3 was significantly lower in prostate cancer tissue when compared to distant healthy tissue, with a decrease also observed in the macroscopically healthy adjacent tissue located near the tumour (Figure 1). Considering that a hallmark of tumourigenesis is stimulated and uncontrolled tissue growth, it is logical to assume that genomic reprogramming would favour apoptosis suppression, particularly through the downregulation of executor caspases such as caspase-3. Supporting this hypothesis, evidence suggests that genomic reprogramming of the healthy cells towards apoptosis suppression leads to uncontrolled proliferation. Apoptosis involves various cellular signalling mechanisms, and in this context, malignant tissue can develop multiple strategies to evade apoptosis. This not only promotes the survival of malignant cells but also increases resistance to antineoplastic drugs, ultimately contributing to disease progression.

The cysteine protease 32 protein, known as caspase-3, has been studied in various modalities of prostate cancer [23] [24] [25]. However, literature data indicate that genomic reprogramming accompanied by gene-programmed silencing of caspase-3 has not been documented in prostate cancer to date [26] [27]. There are even conflicting reports suggesting that increased caspase-3 expression in prostate and breast cancer correlates with cancer aggressiveness [28] [29] [30]. In certain epithelial carcinomas, such as mucosal cancer, as well as in acute myeloid leukaemia, increased caspase-3 expression has also been observed [31] [32]. These findings confirm that caspases do not strictly belong to the tumour suppressor protein family. Based on clinical and experimental data, the most plausible conclusion is that the loss of caspase activity is neither a prerequisite nor a trigger for carcinogenesis. So, caspases are not tumour suppressor proteins in the strict sense. On the other hand, post-transcriptional modifications do not exclude the possibility of reduced activity and apoptotic response.

Among the potential alterations that may occur, the following hypothetical mechanisms have been proposed: 1) Alternative splicing leading to the formation of mRNA that produces a caspase protein with reduced functional activity; 2) Increased presence of caspase inhibitors; 3) Inhibition of downstream pathways leading to cell death during caspase activation; 4) Existence of alternative apoptosis pathways that regulate programmed cell death in cancer independently of caspases. Regarding alternative splicing, studies have demonstrated alternative caspase-3 isoforms in colon cancer (Caspase-3s). This protein is truncated at the C-terminal end, containing the catalytically active cysteine residue [33]. Such splicing prevents the synthesis of functionally active caspase-3, even in cases of extremely high gene expression, as observed in certain breast cancers [34]. Notably, specific inhibitors of caspase-3, known as XIAP (X-linked inhibitor of apoptosis protein), have been identified. XIAP contains specialized BIR domains that bind to caspase-3, inhibiting its activity. Despite these inhibitory mechanisms, the presence of apoptotic cells within tumours, as well as DNA fragmentation, has been documented [35]. Unlike caspase-3 concentration, caspase-1 concentration was significantly increased in prostate cancer tissue, as well as in macroscopically healthy tissue adjacent to the tumour, compared to distant healthy tissue (Figure 1). Caspase-1, also known as ICE (interleukin-1 converting enzyme), is an initiator caspase initially identified as the enzyme responsible for generating active IL-1 from its inactive pro-interleukin-1 precursor [36]. The expression of caspase-1 is not uniformly present across different tumour cells. Studies have shown low expression levels in colorectal, ovarian, and prostate cancers [37].

Pyroptosis is characterized by membrane rupture, pore formation, and the release of inflammatory cytokines IL-1β and IL-18, which can further trigger a cascade of inflammatory responses involving other cytokines. During this process, DNA fragmentation, nuclear chromatin condensation, and nuclear shrinkage may also occur. Additionally, a potential mechanism of carcinogenesis consists of the inactivation of tumour suppressor genes, genomic instability, replication errors, microsatellite instability, and telomere shortening. DNA repair mechanisms, including nucleotide excision repair (NER), base excision repair (BER), and DNA mismatch repair (MMR), are also impaired. These processes are further supported by increased production of free radicals, which have been detected in prostate cancer [38] [39] [40] [41] [42]. It has been shown that, in prostate tissue, caspase-1 triggers transforming growth factor beta (TGF-β)-induced apoptosis. However, high expression of caspase-1 and activation of IL-1β are frequently observed in melanoma, mesothelioma, and epithelial cancers of the digestive tract, promoting carcinogenesis. Experimental data from breast cancer models also suggest that IL-1β activation is strongly associated with tumour cell progression and metastasis [43]. Given the dual role of caspase-1 in carcinogenesis, it is crucial to determine the specific cell type exhibiting high caspase-1 expression. For instance, high expression in inflammatory cells promotes carcinogenesis, whereas high expression in antigen-presenting cells suppresses it. The results obtained in this study may be explained by the coincidence of prostate cancer occurrence with inflammation in the peripheral zone of the prostate. This coexistence strongly supports the notion that inflammation often precedes cancer development. Inflammation can promote tumour growth by inducing DNA damage, usually linked to inadequate DNA repair. Conversely, inflammation can also activate immune defence cells, potentially protecting tissues from malignant cells carrying DNA damage [44] [45] [46] [47] [48].

In the conducted study, alkaline DNase activity significantly increased (nearly threefold) in prostate cancer tissue and the adjacent macroscopically healthy tissue compared to the activity in distant healthy tissue. This increase was observed in both total and specific enzymatic activity. Most immunohistochemical studies conducted in the 1970s demonstrated reduced DNase expression in cancerous tissues and premalignant lesions, including kidney, colon, and liver cancers. Based on these findings, a hypothesis emerged that alkaline DNase expression negatively correlates with cancer development [49] [50] [51]. However, the landscape of cancer biology is far more complex. It has been shown that alkaline and acid DNase activity is markedly increased in malignant tissues that, due to inadequate nutrient supply during proliferation, reach a necrotic stage. Necrosis of the tissue is one of the processes that end in cancer. Paradoxically, the reactivation of endonucleases occurs due to the abundance of necrotic foci. It is believed that the release of enzymes from complexes with inhibitors is a key phenomenon in this process [52] [53]. This has also been confirmed in the case of poly(A) deadenylase in prostate cancer [54]. Regarding enzyme activity in the serum of cancer patients, a significant increase in serum DNase activity has been observed in lung cancer, with a notable decrease following therapy.

Changes in the levels of these markers may have clinical significance in assessing the effectiveness of therapies that induce apoptosis. Reduced expression may be associated with therapy resistance and a more aggressive tumour phenotype. Increased concentration of caspase-1 contributes to the inflammatory tumour microenvironment. Its expression may influence prostate cancer progression, as well as the therapeutic response, particularly in the context of immuno-oncological approaches. Furthermore, further studies with a larger number of patients are necessary for these markers to potentially serve as prognostic indicators of disease aggressiveness and, eventually, theranostic markers.

### The limitations and strengths

Our study is a pilot study. We examined the expressions of caspase-3, caspase-1, and alkaline DNase in prostate cancer patients. Studies addressing other caspase cascades and intracellular calcium levels need to support the current study’s results.

## Conclusion

The concentration of caspase-3 was significantly reduced in prostate cancer, while the concentration of caspase-1 and the activity of alkaline DNase were markedly increased. Given that caspase-3 is considered an executioner caspase in the activation of DNase and DNA fragmentation, these findings suggest that their interdependence is lost, either due to the presence of necrosis or the release of enzymes from complexes with inhibitors. The data obtained regarding caspase-1 imply that the inflammatory response may play a role as a promoter of carcinogenesis.

## Dodatak

### Acknowledgements

This work was supported by the Ministry of Science and Technological Development, Republic of Serbia (Project 451-03-137/2025-03/200113), and Serbian Academy of Sciences and Arts, Nis branch (Projects O-06-17 and -28-22).

### Conflict of interest statement

All the authors declare that they have no conflict of interest in this work.

### List of abbreviations

PC, prostate cancer;<br>PAMP, pathogenassociated molecular patterns;<br>DAMP, damage-associated molecular patterns;<br>ROS, reactive oxygen species;<br>Dnases, deoxyribonucleases;<br>PSA, prostate-specific antigen;<br>XIAP, X-linked inhibitor of apoptosis protein;<br>ICE, interleukin-1 converting enzyme.
